# Palaeozoic giant dragonflies were hawker predators

**DOI:** 10.1038/s41598-018-30629-w

**Published:** 2018-08-14

**Authors:** André Nel, Jakub Prokop, Martina Pecharová, Michael S. Engel, Romain Garrouste

**Affiliations:** 1 Institut de Systématique, Evolution, Biodiversité, ISYEB, UMR 7205, CNRS, MNHN, UPMC, EPHE, Muséum national d’Histoire naturelle, Sorbonne Universités, 57 rue Cuvier, CP 50, Entomologie, F-75005 Paris, France; 2 Department of Zoology, Faculty of Science, Charles University, Viničná 7, CZ-128 00 Praha 2, Czech Republic; 3 Division of Entomology, Natural History Museum, and Department of Ecology & Evolutionary Biology, 1501 Crestline Drive – Suite 140, University of Kansas, Lawrence, Kansas 66045-4415 USA; 4 Division of Invertebrate Zoology, American Museum of Natural History, Central Park West at 79th Street, New York, New York 10024-5192 USA

## Abstract

The largest insects to have ever lived were the giant meganeurids of the Late Palaeozoic, ancient stem relatives of our modern dragonflies. With wingspans up to 71 cm, these iconic insects have been the subject of varied documentaries on Palaeozoic life, depicting them as patrolling for prey through coal swamp forests amid giant lycopsids, and cordaites. Such reconstructions are speculative as few definitive details of giant dragonfly biology are known. Most specimens of giant dragonflies are known from wings or isolated elements, but *Meganeurites gracilipes* preserves critical body structures, most notably those of the head. Here we show that it is unlikely it thrived in densely forested environments where its elongate wings would have become easily damaged. Instead, the species lived in more open habitats and possessed greatly enlarged compound eyes. These were dorsally hypertrophied, a specialization for long-distance vision above the animal in flight, a trait convergent with modern hawker dragonflies. Sturdy mandibles with acute teeth, strong spines on tibiae and tarsi, and a pronounced thoracic skewness are identical to those specializations used by dragonflies in capturing prey while in flight. The Palaeozoic Odonatoptera thus exhibited considerable morphological specializations associated with behaviours attributable to ‘hawkers’ or ‘perchers’ among extant Odonata.

## Introduction

**Figure 1:**
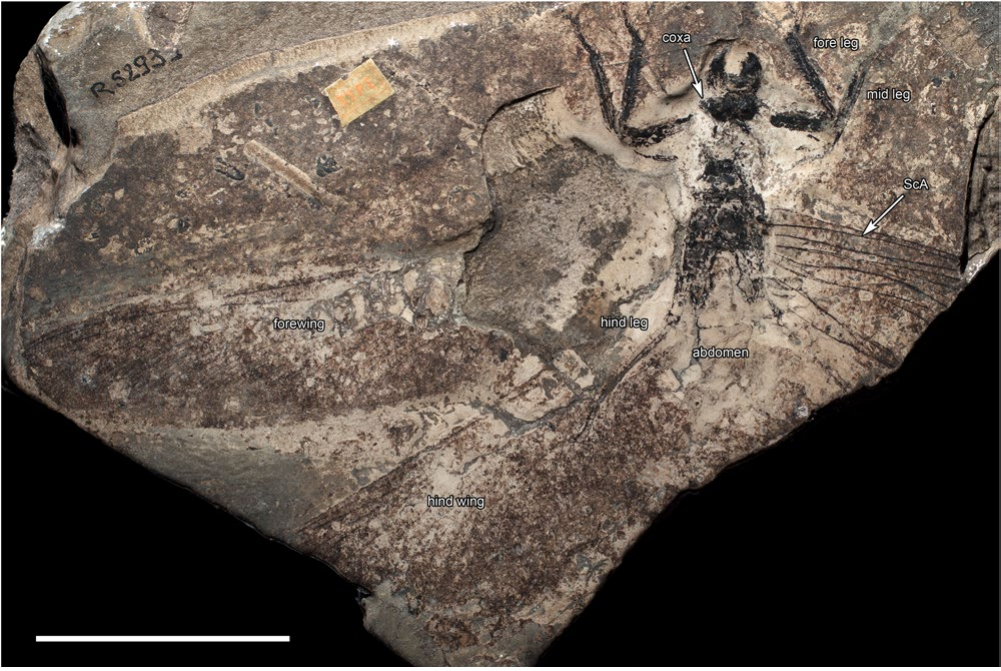
*Meganeurula selysii* (Brongniart, 1893), holotype MNHN R52939, head and prothorax. Ventral view showing the false ‘mandibles’ carved in matrix (photograph Gaelle Doitteau, e-recolnat Project, MNHN). Scale bar, 10 mm.

Despite rampant speculation as to the biology of gigantic insects from the Late Palaeozoic, particularly assumptions regarding their presumed predatory feeding and hunting behaviours, the most pertinent details regarding morphology of Meganeuridae are essentially unknown. Owing to their relationship to modern dragonflies and damselflies (Odonata), many details of odonate biology have been extended to the stem superorder Odonatoptera. Most critically, the anatomical details of the meganeurid head — especially their mouthparts and compound eyes — are effectively unknown. Although *Meganeura monyi* is the iconic giant dragonfly, it is poorly preserved. Only two prior fossils have been reported preserving such portions of giant dragonfly anatomy, viz. *Meganeurula selysii* in which the head and thorax are unfortunately artefacts produced by over preparation (Fig. 1, Extended Data Fig. 1); these, *Namurotypus sippeli* where the head is exceptionally poorly preserved, and *Erasipteroides valentini* revealing little more than the separation of the compound eyes (Supplementary Information), have formed the basis for reconstructions^1^. However, a third fossil, the holotype of *Meganeurites gracilipes* from the well-known Gzhelian outcrop of Commentry (France), has well-preserved head structures which have been long-ignored and therefore never studied or considered in regard to the palaeobiology of these giants. The animal was preserved in fine-grained micaceous sandstone and compressed during fossilization (Supplementary Information). The local palaeoenvironment, approximately 300 Ma (Stephanian B/C), corresponds to a Pennsylvanian limnic biotope in an intramontane basin adjacent to moorland facies^2^.

## Results

(see Supplementary Information for a detailed description of *Meganeurites*).

**Figure 2:**
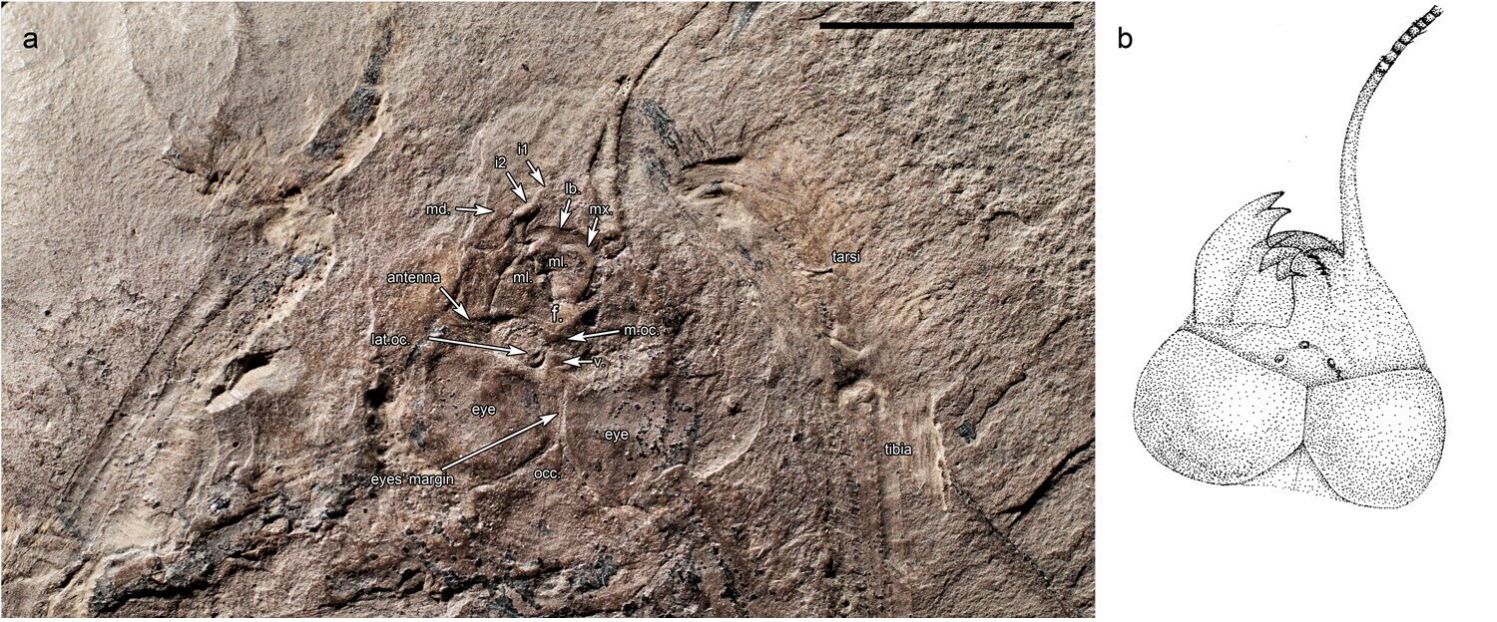
*Meganeurites gracilipes* Handlirsch, 1919, holotype MNHN R53005, head and fore leg. (**a**) dorsal view; (**b**) reconstruction. f. frons, i1, i2 incisivum, lb. labrum, lat.oc. lateral ocellus, md. mandible, ml. molar plate, m.oc. median ocellus, mx. Maxilla, occ. occipital triangle, v. vertex (photograph Gaelle Doitteau, e-recolnat Project, MNHN, reconstruction M.P.). Scale bar, 10 mm.

**Figure 3:**
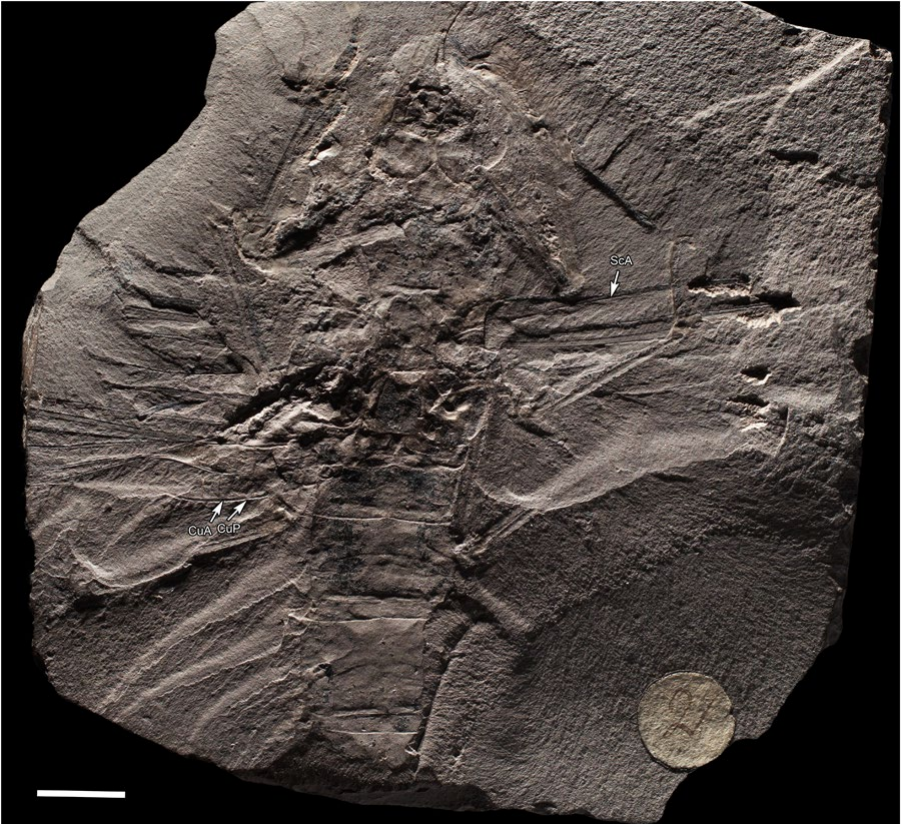
*Meganeurites gracilipes* Handlirsch, 1919, holotype MNHN R53005, general habitus. CuA cubitus anterior, CuP cubitus posterior, ScA subcostal anterior (photograph Gaelle Doitteau, e-recolnat Project, MNHN). Scale bar, 10 mm.

The holotype of *M*. *gracilipes* is fairly complete, certainly by the standards of most known material of meganeurids, with the head, thorax, and much of the legs, wings, and basal part of the abdomen complete, albeit compressed. The counter-part (MNHN.F.R53005) preserves the body in dorsal view (Figs 2 and 3), while the original print, preserving the ventral view of the insect, was lost long ago. The mandibles are prominently preserved and were strong, robust, and with large, sharply acute teeth, similar to those of extant Odonata (Fig. 2). The mandibular form and dentition demonstrates that *M*. *gracilipes*, and likely all species of the family Meganeuridae, were predators. The short antennae with a flagellum of *Meganeurites* probably had the same function in flight control as those of extant Odonata^3^. The positions of the three simple eyes (ocelli) on vertex are in the same positions as those of the extant dragonfly family Aeshnidae, supporting similar roles as the horizon detectors which contribute to the balance body control during fast flight maneuvers^4^.

As in Odonata, the thorax is slanted caudally, resulting in what is referred to as “thoracic skew”^5^, resulting in a slight oblique orientation to the dorsal surface of the thorax and thereby angling the plane of the wings relative to the longitudinal axis of the body. This skew was likely important for Meganeuridae, as it results in an anterior displacement of the legs in *M. gracilipes*, *Meganeurula selysii* (Figs 1 and 3, Extended Data Fig. 1), and Odonata. The more forward position of the legs makes grasping objects in front of the animal easier, as well as the manipulation of materials held in front of the head, such as a prey item. In addition, the presence of strong spines on the tibiae and tarsi, previously known for *Meganeura monyi*^6,7^, and present also in *M*. *gracilipes*, indicates that together the thorax and legs of *Meganeurites* functioned as a”flying trap” for the capture of prey, a morphological and behavioural suite identical to that of modern dragonflies and damselflies.

**Figure 4:**
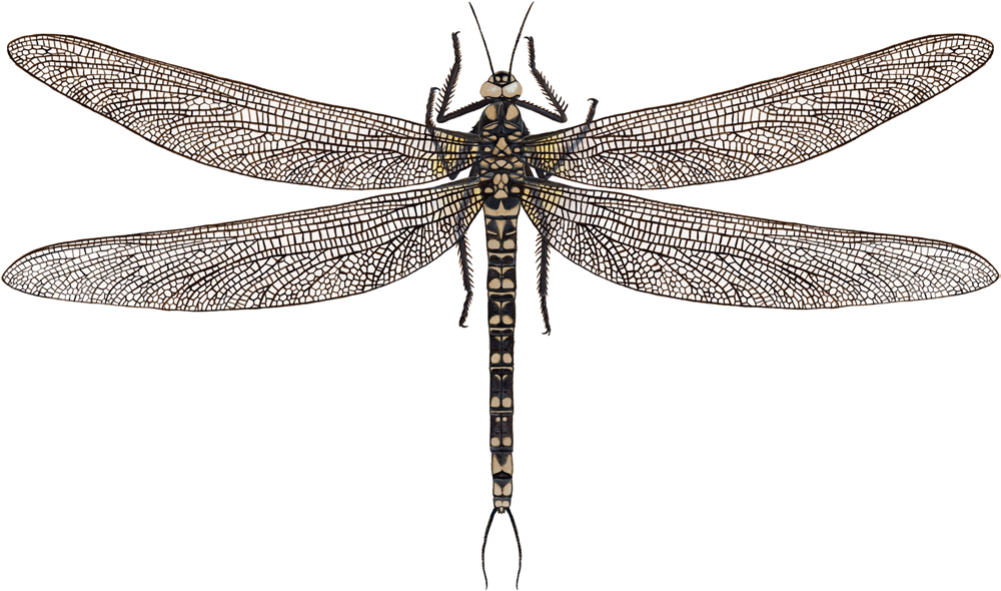
*Meganeurites gracilipes* Handlirsch, 1919, reconstruction. Pattern of coloration highly hypothetical, adapted from extant relatives, caudal appendages of abdomen corresponding to *Namurotypus sippeli* (reconstruction M.P.). Scale bar, 10 mm.

Unlike more basal, earlier-diverging Odonatoptera, *Meganeurites* has no trace of paranotal expansions (‘pronotal lobes’), or prothoracic winglets (Supplementary Information), and it is likely that such an absence is characteristic of all Meganeuridae. Accordingly, meganeurid flight was more similar to those of extant dragonflies than to those of any coeval “six-winged” Palaeodictyoptera^8,9^. Nevertheless, the absence of nodal flexion structure in Meganeuridae probably prevented them from achieving flight performances similar to those of the true Odonata, viz. with the capacity to twist and make abrupt, directional changes while in flight^10^. Accordingly, *Meganeurites* was more likely an open-space, ecotone, or riparian forest predator. Using modern odonates as an analogue, meganeurids would have been ‘hawkers’, patrolling above large rivers, ancient lakes, open forests, or even above the canopy^11,12^, rather than ‘perchers’, who fly in rapid, zig-zag formations through relatively dense forest environments, with liana-like foliage already present during the Ghezlian^13,14^. In the latter habitats, the large wingspan of *M*. *gracilipes* (ca. 320 mm) would have been a significant handicap to fly or even glide (Fig. 4). As is true for modern hawker dragonflies, excellent visual acuity is critical for catching large, flying preys. This would have been true for *M*. *gracilipes*. These large Meganeuridae were probably preying on large Palaeodictyoptera also present in the palaeobiota of Commentry.

Visual precision would also have been critical for *Meganeurites* to evade its own predators which were probably larger meganeurids, such as *Meganeura monyi* with its wingspan of ca. 700 mm, characteristic of the fauna at Commentry. Flying and gliding vertebrates appeared ca. 30 myr later. Similar preying behaviours are known for extant Anisoptera^15^. *Meganeurites* had enlarged compound eyes with broad dorsal portions, meeting medially for significant portion of their length, as in extant hawker dragonflies (Fig. 2, Extended Data Fig. 2). Among all fossil and extant Odonatoptera, only the Aeshnidae and some ‘libelluloids’ (Macromiidae, some ‘Corduliidae’, and Pantaliinae and Zyxommatinae among Libellulidae) have dorsally meeting compound eyes^16–18^, and are ‘hawkers’, “remaining in flight continuously throughout the day, and foraging in flight by swooping up to grab insects passing overhead”^19^. Owing to the hypertrophied development of the dorsal portion of the compound eyes and likely specialized ommatidia associated with such a condition, such dragonflies can more easily detect objects (prey or predators) against the blue sky^20–22^. We can infer from the eyes’ shape that the condition was similar for *Meganeurites* and that it would have also had excellent vision, consistent with the ‘hawker’ behaviour implied by the size of its wings. Extant Aeshnidae have specialized ommatidia on dorsal part of the eyes, but, unfortunately, the ommatidia are not preserved in the holotype of *Meganeurites*. Many of those extant taxa with large and broadly confluent compound eyes in dorsal view are crepuscular, such as Zyxommatinae and *Tholymis* (Libellulidae), *Apomacromia* (Corduliidae), *Aeshna viridis*, *Limnetron* (both Aeshnidae), or Gynacanthinae (Extended Data Fig. 2)^16,23–26^. This is not the case for all dragonflies with broadly confluent compound eyes, and some crepuscular gomphids have separated compound eyes. Accordingly, it is not possible to say with certainty that *Meganeurites* was similarly crepuscular, although this was most likely. The head of *Meganeurites* was narrower than the thorax (Figs 3 and 4), suggesting that it is possible that its vision was less optimal than that of modern Anisoptera with large compound eyes. The presence of dorsally adjoining eyes is a potential apomorphy of the Meganeuridae (or the subfamily Tupinae), to be verified through the discovery of new fossils spanning the diversity of this group. The widely separated compound eyes of the giant *Erasipteroides valentini* (Erasipteridae)^27^ led previous authors to consider erroneously that this was characteristic of all giant dragonflies^28^. On the contrary, the morphological disparity of the compound eyes among Palaeozoic meganeurids was apparently as important as it is today among modern dragonflies (Supplementary Information), suggesting a similar behavioural diversity among these early odonatopterans.

We performed two multivariate morphometric analyses on four fossil taxa and 21 extant Odonata in regards to the morphology of the head, forelegs, thorax, and wings (Supplementary Information). The considerably large sizes of the chosen Carboniferous and Jurassic Odonatoptera have a great influence on the results (Extended Data Fig. 4). Nevertheless, the observation along axes 2 and 3 of the first analysis (raw data) minimizes the effect of size and demonstrates that *Meganeurites* falls among the extant hawkers (Extended Data Fig. 5). We show that the false ‘head’ of *Meganeurula selysii* and the rather poor preservation of the head of *Erasipteroides valentini* place them well apart from all other taxa (Extended Data Figs 4–6).

During the Late Carboniferous, there simultaneously existed comparatively small, damselfly-like Odonatoptera^29,30^, probably living in densely forested environments, catching small prey along rivers or within the forests themselves. Later, during the Permian, the Meganeuridae also diversified into a range of taxa, spanning sizes from gigantic to species whose wingspans more closely approximate those of extant Anisoptera^7^, and confirming the co-existence of different life habits for these ancient flying predators. The discovery of evidence relating to the palaeobiology of the charismatic, iconic, and giant dragonflies also reveals a remarkable consistency in predatory biology, as well as a range of variants known still today, along the odonatopteran lineage over the course of at least 300 million years.

## Methods

### Material studied

The two specimens MNHN.F.R53005 and MNHN R52939 are deposited in the collection of Palaeontology, MNHN, Paris, France.

### Observation and description

Photographs were taken with a Nikon D800 digital camera with AF-MicroNikkor 60 mm, using four optical fibers to improve the light. Original photographs were processed using the image-editing software Adobe Photoshop CS6. Standard wing venation nomenclature is followed for Odonatoptera^31,32^. Two Principal Component Analysis were performed to compare the morphology of the fossil and extant Odonatoptera (Supplementary Information).

### Online Content

Additional Methods, Extended Data display items and Source Data are available in the online version of the paper; references unique to these sections appear only in the online paper.

## Electronic supplementary material

Supplementary Material

## Data Availability

All data generated or analyzed during this study are included in this published article (and its Supplementary Information files).
