# Supplementary material for: Palaeozoic giant dragonflies were hawker predators
Source: Sci Rep. 2018 Aug 14;8:12141. doi: 10.1038/s41598-018-30629-w (PMC6092361; doi:10.1038/s41598-018-30629-w)

**Supplementary Information**

# Palaeozoic giant dragonflies were hawker predators

André Nel, Jakub Prokop, Martina Pecharová, Michael S. Engel and Romain Garrouste

## Revision of the genus *Meganeurites* (Odonatoptera: Meganeuridae)

### Material and methods

The fossils were examined under binocular microscope Nikon SMZ 1500 and photographs were taken using a camera Nikon D800, under alcohol and dry. We follow the meganisopteran classification of Nel et al.[1], for venation nomenclature, see Bechly[2].

### Description

Family Meganeuridae Handlirsch, 1906

Subfamily Tupinae Handlirsch, 1919

Genus *Meganeurites* Handlirsch, 1919 stat. rest., nov. sit.

Type species. *Meganeurites gracilipes* Handlirsch, 1919 (comb. rest.).

*Meganeurites gracilipes* Handlirsch, 1919 (comb. rest.)

Figs 2-4

1909 *Meganeurula selysii* - Meunier, pl. 4, fig. 2

1919 *Meganeurites gracilipes* Handlirsch, 570, fig. 69 (original description)

1943 *Meganeurula selysii* - Carpenter, 544-546, pl. 1, fig. 2 (revision and synonymy)

1949a *Meganeurula selysii*? - Carpentier & Lejeune-Carpentier, 317-325, pl. 1 (description, abdomen morphology)

1949b *Meganeurula selysii* - Carpentier & Lejeune-Carpentier, 553-554 (description, head morphology)

1952 *Meganeurula selysii* - Carpentier, 161 (morphology of the thorax)

1953 *Meganeurula gracilipes* - Carpentier, 183-184 (morphology of the thorax)

Material. Holotype MNHN.F.R53005 (counter-part), stored at the Laboratory of palaeontology, MNHN, Paris. The part should be stored at the same place but it is currently lost, and was perhaps not returned by Carpentier who was the last researcher to study it.

Remarks. *Meganeurites gracilipes* has a long taxonomic history. Meunier[3], in his pl. 4, fig. 2, first figured the part of the holotype under the name '*Meganeurula selysii*' (Brongniart, 1893) without describing it. Handlirsch[4] described the genus and species as *Meganeurites gracilipes*, on the basis of Meunier's photograph, but without seeing the specimen. Carpentier and Lejeune-Carpentier[5-7] and Carpentier[8] restudied the part, while Carpenter[9] (pl.1, fig. 2) also re-studied the counter-part. A comparison of the positions of the legs in Carpenter's and Carpentier's figures and Fig. 4 is sufficient to demonstrate this point. The part studied by Carpentier is currently lost.

Age and outcrop. Gzhelian, Commentry, Allier, France.

Preservation. The part preserved the body with the apex of the abdomen missing, nearly complete legs, and a nearly complete wing[3]. The counter-part preserves the body with legs and wing bases. The fossil is clearly compressed, and the whole body is probably deformed, too broad. The abdomen is 14.7 mm wide, while *Arctotypus intermedius* Nel et al., 2009 from the Permian of Lodève, fossilized in volume with very few deformations, has an abdomen 10 mm wide with the same wing length ca. 150 mm; another undescribed Meganeuridae from the same outcrop has a wing length > 150 mm and an abdomen ca. 12 mm wide[1]. The measurements only give an idea of the original dimensions of the head, thorax, and abdominal

length and width. There are some traces of pin marks around the left ocelli and antenna, but none are visible around the mandibles and compound eyes.

Redescription. Head 14.4 mm long, 13.5 mm wide; compound eye rounded from above, 6.7 mm long, 6.7 mm wide; compound eyes meeting for 3.1 mm; vertex small, rectangular, 0.9 mm long, 1.5 mm wide; lateral ocellus 0.8 mm wide; median ocellus 0.8 mm wide; frons (+ clypeus?) 3.4 mm wide, ca. 2.0 mm long; general shape of vertex, lateral and median ocelli, and bases of antennae similar to those of extant Aeshnidae, due to broadly confluent eyes; mandibles strong, 6.7 mm long, 3.1 mm wide, with first incisor 1.7 mm long, second incisor 0.9 mm long, molar ca. 1.0 mm wide, with two visible strong teeth; space between second incisor and molar space narrow, similar to situation in extant Aeshnidae, while other modern Anisoptera have a broader space[10]; labrum displaced, visible in front of left mandible, apparently covered with small granulations; frons strongly deformed, visible between vertex and mandibles.

Legs: Tarsi tetramerous, tarsomeres with strong ventral spines, showing two strong apical teeth; all tibiae with a double row of strong spines; profemur ca. 17.8 mm long, protibia 17.7 mm long, protarsus ca. 12.7 mm long; mesotibia 17.7 mm long. Basal parts of legs hidden below thorax (dorsal view), while coxae visible (on part figured in Meunier[3], ventral view). [Thoracic skewness is important as the legs are clearly displaced anteriorly relatively to the bases of wings, as in extant dragonflies].

Wings. Only basal parts preserved. Visible venation typical of Meganeuridae. ScA short, 15.3 mm long; transverse veins CuP and CuA well-visible between M+Cu and AA, oblique and well separated.

Abdomen incomplete, showing four segments at most, preserved part ca. 33.4 mm long, 14.7 mm wide; segment I 5.3 mm long, segment II 10.0 mm long, segment III 11.7 mm long, segment IV 6.4 mm long (as preserved) but incomplete.

## Discussion

Taxonomy

*Meganeurula selysii* has an elongate ScA and a broad 'subcostal' area between the costal margin and ScA (Fig. 1), at least in the forewing, which is a synapomorphy of the Meganeurinae[1], while MNHN.F.R53005 has no long ScA and the 'subcostal' area between the costal margin and ScA is short and narrow (Fig. 3). Also, this last fossil clearly preserves the braces CuP and CuA oblique and separated, which are tupine characters. Thus, *M. gracilipes* is clearly different from *Meganeurula selysii*. Also, *M. gracilipes* does not fit in Piesbergtupinae because of its distinctly broader area between AA and AP[11]. We restore the genus *Meganeurites* and transfer *M. gracilipes* in the Tupinae (nov. sit.).

*Gilsonia titana* Meunier, 1909 and *Meganeurina confusa* (Handlirsch, 1919) are the two other described Tupinae from Commentry[1]. After Meunier's figure[3], the preserved part of the forewing of MNHN.F.R53005 is 131.2 mm. As it shows parts of RP's branches, the complete wing can be estimated around 150 mm. The holotype of *G. titana* has also an incomplete forewing, but ending nearly at the same point as for *M. gracilipes*; and its preserved part is only 109 mm long. Thus, *G. titana* has wings shorter than *M. gracilipes*, and corresponds to a different species. The holotype of *M. confusa* also has incomplete wings, but the preserved part ends well basal of that of *M. gracilipes*, and is already 131 mm long, thus the complete wing was certainly longer than that of *M. gracilipes*. They probably also correspond to different taxa. The incompleteness of all these fossils, especially in their wing venation, prevents us from being more accurate of their generic distinctions. While they have been put in different genera, precise distinctions between these genera remain questionable. Nevertheless, in the absence of evidence to the contrary, we have maintained these as distinct genera.

Morphology

- Head: Carpentier and Lejeune-Carpentier[6] indicated that the head bears two large contiguous structures ('deux grosses masses contiguës') that they refused to consider as the compound eyes. As these authors had in their hands the ventral part of body showing the legs' insertions, these 'masses' are not so well-preserved as on the counter-part that preserves a dorsal view of the body. These structures are in the exact position of the compound eyes. These are dorsally meeting for quite a long distance (Fig. 1). The reconstructions[12-13] of the tupine *Namurotypus sippeli* Brauckmann & Zessin, 1989 exhibit a head with the compound eyes widely separated, but the original photographs of this taxon[14-15] clearly show that the head structures of *N. sippeli* are poorly preserved, with the compound eyes not visible.

Few odonatopteran clades have the compound eyes meeting or fused dorsally. It is the case for the extant Aeshnidae and some Cavilabiata, especially in Macromiidae and Libellulidae, Pantaliinae, and Zyxommatinae. Macromiids (or cruiser dragonflies), aeshnids (or hawkers), pantaliines, and zygommatines today hunt over the surface of bodies of water. The dorsally fused compound eyes of the Libellulidae and Aeshnidae are of different shapes (Extended Data Fig. 2)[16], and were convergently acquired, with differences in the structure of the suture between the compound eyes[17]. Thus, at least some Meganeuridae also convergently acquired similar compound eyes.

- Thorax. Carpentier[8] indicated the presence of large paranotal expansions on specimen R.53005, which are absolutely not visible. He has probably confused a depression in the matrix with such structures. Carpentier & Lejeune-Carpentier[7] also cited the presence of large paranota on the thorax of the holotype of *Meganeurula selysii* (specimen MNHN.F.R5293), but as indicated above this specimen has been in great part destroyed by preparation made a long time ago, so that it is impossible to have any accurate indication of the original

morphology of the thorax and head (Extended Data Fig. 1). As there are no traces of paranotal expansions, pronotal lobes, or of pronotal winglets on MNHN.F.R53005, the Meganeuridae probably did not have any such structures, unlike the putatively 'basal' Odonatoptera like *Argentinala*[18].

The thoracic skewness was probably rather important in the Meganeuridae, as it can be seen after the anterior displacement of the legs of MNHN.F.R53005 and MNHN.F.R52939 (Figs. 1–3).

- Legs. The presence of strong spines on tibiae and tarsi strongly suggests that the legs of MNHN.F.R53005 had a function of 'flying trap' to capture prey, as is the case for extant Odonata. MNHN.F.R53005 also confirms that the Meganeuridae, as already known for *Meganeura monyi*, had pentamerous tarsi[19], while *N. sippeli* purportedly has tetramerous tarsi[13-14]. The reduction of the number of tarsomeres in Tupinae could be a synapomorphy of this clade. Zessin[12] indicated the presence of three pretarsal claws for *N. sippeli*, which is quite strange for an insect, where the claws are exclusively paired or sometimes with one or both reduced. We could only observe the two 'normal' claws in MNHN.F.R53005 as in the type of *M. monyi*, also present in extant Odonata. The situation in *N. sippeli* should be verified and might be a misinterpretation.

- Abdomen. Carpentier & Lejeune-Carpentier[5-6] described some lateral abdominal 'lanières à extrémité effilées' for *Meganeurites*. Carpentier[8] added that the lateral abdominal 'filaments' are not submarginal. In fact, these structures are clearly visible (Fig. 4), but they are not organic but simple breaks in the matrix. This fossil has no lateral filaments (gills?) at all. The Meganeuridae had no lateral abdominal appendages, as previously indicated[1] on the basis of the study of Permian Tupinae. Also, the same authors interpreted some breaks in the matrix at the end of the preserved part of the abdomen as strange abdominal appendages. The abdomen of MNHN.F.R53005 is incomplete (Fig. 3), with only four segments preserved. It is highly

probable that the apical part of the abdomen was hidden under the matrix of the part, as it is visible in the photograph of Meunier[3]. Even if we have little information on the structure of the abdomen of these insects, they had 10 abdominal segments as in extant Odonata. MNHN.F.R53005 has no visible secondary male genital apparatus on the second abdominal segment, but this segment is visible in dorsal view so such structures could be hidden. It could also be a female or it had a manner of mating different from that of the Odonata, as already supposed[13]. The oldest Odonatoptera with a visible male secondary genital apparatus are Permian and belong to the grade 'Protozygoptera'[19].

## Statistical analysis of recent and fossil dragonfly morphometrics

### Material and methods

We performed two PCA (Principal Component Analysis), a multivariate method conventionally used in morphometry and biometrics[20] widely used in zoology, botany, and evolutionary biology. We analyzed and visualized a dataset corresponding to measurements taken from four fossil taxa and 21 extant Odonata. These were chosen for their representativeness of the sizes of bodies, wings, and eyes among extant Odonata. The studies are based on 12 measurements of the head (compound eyes), thorax (Th), prothoracic legs (P1), and wings (Extended data fig. 3).

These measurements were taken with vernier calipers (0.1 mm precision) on collection specimens (Arthropods Collections, MNHN Paris) and have been preferred to automated methods that are sources of error. Asymmetry was not considered; all measurements were done on the right side, by default. Sex was not considered in this analysis because it is not frequently known for all compression fossils, but it needs to be considered in studies of extant taxa (sexual dimorphism vs. polymorphism). The measurements made are the smallest

denominator between the fossil and the extant specimens. The final dataset has no missing data. The list of taxa measured is given in Supplementary Table 1.

**Supplementary Table 1 | List of studied Odonatoptera.** Fossils in bold, 1 specimen per taxon (MNHN collection)

| Families | Species |
|---|---|
| Aeshnidae (Aesh) | *Gynacantha gracilis* Burmeister, 1839 |
| Aeshnidae | *Aeshna viridis* Eversmann, 1836 |
| Argiolestidae (Arg) | *Caledopteryx maculata* Winstanley & Davies, 1982 |
| Calopterygidae(Calop) | *Calopteryx cornelia* Selys 1853 |
| Calopterygidae | *Calopteryx virgo* (L., 1758) |
| **Campterophlebiidae (Camp)** | ***Bellabrunetia catherinae*** Fleck & Nel, 2002 **(Belcat)** |
| Coenagrionidae(Coena) | *Epipleoneura capilliformis* (Selys, 1886) |
| Cordulegastridae (Cordule) | *Cordulegaster boltonii* (Donovan, 1807) |
| Corduliidae (Corduli) | *Oxygastra curtisii* (Dale, 1834) |
| Epiophlebiidae (Epiop) | *Epiophlebia superstes* (Selys, 1889) |
| **Erasipteridae (Era)** | ***Eraspiteroides valentini* (Eraval)** |
| Gomphidae (Gomph) | *Phyllogomphus perisi* (Compte Sart, 1963) |
| Gomphidae | *Lindenia tetraphylla* (Vander Linden, 1825) |
| Libellulidae (Libell) | *Tetrathemys bifida* Fraser, 1941 |
| Libellulidae | *Tramea rustica* De Marmels & Rácenis, 1982 |
| Libellulidae | *Zygonix torridus* (Kirby, 1889) |
| Libellulidae | *Tholymis tillarga* (Fabricius, 1789) |
| Macromiidae (Macro) | *Macromia congolica* (Fraser, 1955) |

| | |
|---|---|
| **Meganeuridae (Meg)** | ***Meganeurites gracilipes* (Meg)** |
| **Meganeuridae** | **Meganeurites gracilipes* +10 **(Meg+)** |
| **Meganeuridae** | **Meganeurites gracilipes* -10 **(Meg-)** |
| **Meganeuridae** | ***Meganeulula selyssi*** (artefact) **(MegF)** |
| Petaluridae (Peta) | *Petalura gigantea* (Leach, 1815) |
| Petaluridae | *Uropetala carovei* (White, 1846) |
| Pseudostigmatidae (Pseud) | *Megaloprepus caerulatus* (Drury, 1782) |
| Pseudostigmatidae | *Mecistogaster amalia* (Burmeister, 1839) |

For *Erasipteroides* and the Middle Jurassic *Bellabrunetia*, the measures come from the original descriptions[21-22]. To eliminate the effects of taphonomic and tectonic deformation, we considered two additional data represented by [Meg+] and [Meg-] measurements for *M. gracilipes*. These are the measures of [Meg] increased (Meg+) or minus (Meg-) by 10%.

The studied extant species cover several families from the two main odonatan suborders, Epiproctophora (damsel-dragonflies and dragonflies) and Zygoptera (damselflies) (Supplementary Table 2). The largest representatives (wingspan) of the extant Odonata have been taken into account (Petaluridae and Pseudotigmatidae). Hunting behaviors are taken into account, H (hawker) for hunters in flight, and P (perching) for species that hunt from a support or catch prey that do not fly. Some species are also known for their twilight behavior, as in the genus *Gynacantha* (Libellulidae).

**Supplementary Table 2 | Measurements for morphometric analyses.**

**Table 2**

**Morphometric measurements (Odonatoptera)** see abbreviation (abbr.) in text

| families | taxa | abbr. | Th1w | Thmax | HW | Fem1L | Fem1w | Ti1L | Ti1w | Ey-con | EE-Dist | WinS | Win1L | Win1w | behavior |
|---|---|---|---|---|---|---|---|---|---|---|---|---|---|---|---|
| Meg | *Meganeurites gracilipes* | Meg | 14 | 15 | 14 | 20 | 4 | 22 | 3 | 3 | 0 | 320 | 158 | 40 | Meg |
| Meg | *Meganeurites gracilipes +10* | Meg+ | 15.4 | 16.5 | 15.4 | 22 | 4.4 | 24.2 | 3.3 | 3.3 | 0 | 352 | 173.8 | 44 | Meg |
| Meg | *Meganeurites gracilipes-10* | Meg- | 1.6 | 13.5 | 12.6 | 18 | 3.6 | 19.8 | 2.7 | 2.7 | 0 | 288 | 142.2 | 36 | Meg |
| Meg | *Meganeulula s. fake* | Megf | 15 | 12 | 12 | 17 | 6 | 22 | 5 | 0 | 3 | 350 | 170 | 45 | MegF |
| Era | *Eraspiteroides valentini* | Eraval | 7 | 12 | 8 | 5 | 3 | 5 | 2 | 0 | 5 | 160 | 80 | 30 | Eraval |
| Libell | *Tetrathemys bifida* | Tetbi | 4.5 | 4.6 | 5.4 | 4 | 0.8 | 3.5 | 0.5 | 1.8 | 0 | 44 | 23 | 4.8 | H |
| Gomph | *Phyllogomphus perisi* | Phype | 4.4 | 8.6 | 9.5 | 6.5 | 1.9 | 4.5 | 0.9 | 0 | 2.8 | 96 | 43.5 | 9 | P |
| Macro | *Macromia congolica* | Maco | 3.5 | 4.6 | 6.5 | 4.5 | 0.7 | 4.6 | 0.4 | 2.2 | 0 | 59 | 28 | 6.5 | H |
| Libell | *Tramea rustica* | Traru | 2.8 | 6 | 7.5 | 6.5 | 0.9 | 4.8 | 0.3 | 3.2 | 0 | 83 | 39 | 7.8 | H |
| Calop | *Calopteryx cornelia* | Calco | 2.7 | 4.8 | 6.5 | 7.6 | 1 | 8.5 | 0.2 | 0 | 2.9 | 82 | 44 | 13 | P |
| Aesh | *Gynacantha gracilis* | Gyngr | 7.5 | 5 | 11 | 7 | 1 | 8 | 0.5 | 4 | 0 | 111 | 54 | 12 | H |
| Libell | *Zygonix torridus* | Zygto | 4 | 6.5 | 6.9 | 6 | 1.9 | 4.8 | 1.1 | 2.8 | 0 | 100.5 | 51.5 | 12.6 | H |
| Libell | *Tholymis tillarga* | Thoti | 3.9 | 5.1 | 7.5 | 3.9 | 1 | 3.8 | 0.5 | 3.5 | 0 | 72.8 | 36.4 | 8.9 | H |
| Peta | *Petalura gigantea* | Petgi | 9.1 | 11.2 | 13.6 | 12.2 | 2.5 | 10.3 | 0.9 | 0 | 3.8 | 123.4 | 60.6 | 13.8 | H |
| Peta | *Uropetala carovei* | Uroca | 8.5 | 10.2 | 11.5 | 9 | 2.1 | 7.8 | 1.2 | 0 | 2.8 | 114.8 | 55 | 11.9 | H |
| Epiop | *Epiophlebia superstes* | Episu | 3.4 | 5.1 | 7.4 | 5.8 | 0.8 | 4.6 | 0.5 | 0 | 1.1 | 66.2 | 28.9 | 6.5 | P |
| Aesh | *Aeshna viridis* | Aesvi | 4.2 | 8.9 | 9.6 | 5.4 | 0.9 | 6.4 | 0.5 | 4 | 0 | 93.8 | 44.2 | 12.3 | H |
| Pseud | *Megaloprepus caerulatus* | Megco | 4 | 5.6 | 8.9 | 6.2 | 1 | 6.3 | 0.6 | 0 | 3.3 | 162.5 | 87.3 | 20.8 | P |
| Pseud | *Mecistogaster amalia* | Mecam | 2.8 | 4.2 | 6.8 | 4.3 | 0.8 | 4.1 | 0.4 | 0 | 2.8 | 125.2 | 64.7 | 8.5 | P |
| Calop | *Calopteryx virgo* | Calvi | 3 | 4.1 | 5.5 | 4.2 | 0.6 | 6.1 | 0.3 | 0 | 2.9 | 62.3 | 34.1 | 13.5 | P |
| Corduli | *Oxygastra curtisii* | Oxycu | 2.9 | 6 | 7.8 | 4.2 | 1.2 | 3.5 | 0.5 | 1.2 | 0 | 67.4 | 31.2 | 9.8 | P |
| Gomph | *Lindenia tetraphylla* | Linte | 6.5 | 8.5 | 9.2 | 5.7 | 1.5 | 4.8 | 0.6 | 0 | 1.2 | 80.2 | 38.4 | 10.1 | H |
| Megap | *Caledopteryx maculata* | Calma | 2.5 | 3.4 | 6.1 | 4 | 1 | 5.2 | 0.5 | 0 | 2.9 | 82.4 | 39.5 | 7.6 | P |
| Cordule | *Cordulegaster boltonii* | Corbo | 6.2 | 8.5 | 11.1 | 8.2 | 2.1 | 6.1 | 0.8 | 0.5 | 0 | 104.5 | 54.2 | 10.3 | H |
| Coena | *Epipleoneura capilliformis* | Epica | 1.1 | 1.8 | 3.1 | 4 | 0.2 | 3.5 | 0.1 | 0 | 1 | 32.8 | 15.7 | 4.5 | P |
| Camp | *Bellabrunetia catherinae* | Belcat | 10.2 | 12 | 12 | 11 | 3.4 | 13 | 1.8 | 0 | 6 | 198 | 89 | 14.2 | Belcat |

A morphometric PCA allows the construction of a morphospace[23-24] to reduce the data to a theoretical mathematical space constructed from a dataset. Two analyses were conducted (R Software and R-Studio, ADE-4 package[25-26]) (1) the raw dataset analysis without transformations and (2) an analysis on the data transformed into log shape ratio[27], to eliminate this effect and take into account variations of shape. This method consists in subtracting the average per individual from the logs of the measurements to each of the individual measurements. Morphometric measurements are sensitive to size effect. A large part of morphometrics is to overcome this effect to highlight the differences in shapes that can be hidden in allometric relationships[28]. The graphical representations display the projection of principal components (first axis, ranking by inertia, a proxy for total variance) of the individuals (Odonatoptera spp.), the variables in a correlation circle, and the histogram of the eigenvalues (percentage of inertia per axis). In these projections, the individuals belonging to a taxonomic (family) or behavioral (H or P) group are grouped in distribution ellipses (in this case 67%) whose center of gravity is connected to each point ('star'). Dotted lines materialize the main axes of these ellipses. For elements with two taxa, the ellipses are sticks. In all cases, these representations indicate the variability within the morphospace of the constituted group and makes it possible to analyze their positioning in the space (relative position of barycenters and points).

Two complementary tests are used to analyze the significance of the groups discriminated within the morphospace, ANOSIM (analysis of similarity) and ADONIS (Permutational Multivariate Analysis of Variance Using Distance Matrices, a multiple ANOVA method), two tests of the VEGAN package for R[29].The graphic output of R is modified using Adobe Illustrator CS6.

**Results**

The analysis 1 (Extended data fig. 4) makes it possible to visualize the position of the fossils (Meg, MegF, Belcat, Eraval) in the morphospace (axes 1-2) constituted by the spatialized morphometric data set without transformation). The considerably large size of fossil Meganeuridae (Meg but also of MegF) and *Erapsiterites* (Erap) are discriminated against other taxa. In a classical way[28], the size effect is strongly highlighted on axis 1: all the variables except EE.Dist and EY.con are correlated to this axis as shown by the concomitant direction of the variables on the circle of correlations (Extended data fig. 4). This axis also carries a large part of the inertia analysis (85%), which shows the preponderance of this size variable (Extended data fig. 4). Meg, Meg+ and Meg- form a triplet in the morphospace, whose barycenter is the average position (taking into account the taphonomic deformation by this variability range). MegF is associated to this ellipse to show its relative proximity, due to the size effect. Extended data fig. 4 shows the extant taxa grouped by their hunting behavior (H for Hawker and P for percher). Perchers are represented by the Zygoptera (Pseudostigmatidae, Calopterygidae, etc.) that hunt mainly from a support or in a particular way as Pseudostigmatidae that capture spiders in their webs, plus Epiophlebiidae, etc. The other Epiproctophora (e.g., Petaluridae, Libellulidae) are considered as flight hunters (hawkers), some are crepuscular with specialized compound eyes (in Libellulidae or *Gynacantha* sp.). The ANOSIM test and the ADONIS method are both highly significant to explain the partition between fossils (three categories) and the two different hunting behaviors (ANOSIM, ddl = 5, 999 permutations, $P < 0.004$, ADONIS, ddl = 5, 999 permutations, $P < 0.0003$).

Extended data fig. 5 represents the axes 2-3 projection allowing a first approach in order to avoid the size effect without transformation of the data. The Meg group (represented by Meg, Meg+ and Meg-) and the barycenter of the ellipse is clearly close of the cloud of hunters. The

shape of the eyes that determines this proximity by the value of EE-con (eye contiguity), and high eye-eye distance. MegF is not associated to the [Meg, Meg+, Meg-] group because it is a partly an artefact produced by over preparation.

In the log shape ratio analysis (Extended data fig. 6), despite of the transformation, the size, and especially the wing size (Wingspan, wing length and width) strongly influence the positions of large species (*Meganeurites* and *Erapsiteroides*).

**Discussion**

The extant Odonata are good models for studies of morpho-functional evolution of particular organs but it is essentially the wings that are taken into account[30]. Few studies combine other characters than wings in the morphometrics of Odonata.

Even less markedly, fossils are rarely taken into account in this type of study, which is confined to the micro-evolutionary level of population or phylogenetic studies in a restricted set (effects of migration and various life traits, including biomechanics)[31-32]. The insertion of fossils in this type of study is promising and allows morpho-functional inferences as in the case of mimicry in fossil orthopteran wings[33].

The multivariate analysis of 12 morphological variables of 25 Odonatoptera, including four fossil taxa (Extended data figs. 4-5) shows a very strong influence of size at the expense of the shape of the structures that we seek to implement. However, we consider size as an important variable because it has been defined as an apomorphy to characterize the Meganeuridae[2]. Nel et al.[1], when describing Meganeuridae less than or equal to 100 mm in size (wingspan), discussed this trait, which no longer can be accepted as an apomorphy of this family. However, we must consider that the size of these organisms also influences their functional morphology and thus the shape of certain organs. The arrangement of the compound eyes, not much contiguous to broadly contiguous (Ey-con), and their distance

when they are separated (EE-Dist), discriminate quite well two taxonomic sets (Extended data fig. 4). This arrangement also distinguishes the two sets of extant Odonata represented by hawkers (Libellulidae, Petaluridae) and perchers (mainly Zygoptera) (Extended data fig. 5). Meganeuridae are well differentiated in this scheme, approaching the morphology of hawkers (shape of the head and legs, including the length of the protibia). MegF (*Meganeurula selysii*) is divergent from the other Meganeuridae in the context of this study, since this specimen is 'over-prepared' at the level of the head (eyes and especially the mandibles exaggeratedly prominent). Its abnormal position confirms this fact. So it must be removed from analyses and discussion. *Erapsiteron* is well separated from all the other taxa. It is also not well preserved; leading to a problem of interpretation, and must be reconsidered in this sense. The Jurassic fossil *Bellabrunetia* (Belcat) is also quite well separated from other taxa, probably by its size larger than in the extant Odonata. The ANOSIM and ADONIS (a MANOVA) tests confirm the partitions of the morphospace and the relevance of the analysis by a high significance that also confirms the relevance of the dataset (characters and specimens) for this issue. Further analysis with expanded data needs to be performed to improve this approach.

Several ways exist to reduce the size effect in this type of analysis[28]. However, in this first analysis, taking into account axes 2 and 3 (more than 30% of inertia) makes it possible to highlight the conformation (shape) by excluding the component which carries the greatest part of inertia due to the correlation with size (Extended data fig. 5). The distinction of taxa into perching hunters or hawkers makes it possible to distinguish two morphologically differentiated groups (P, H). To avoid influencing the analysis, we did not make a priori assignments for any of the four fossil taxa. In this analysis, *Meganeurites* [Meg] is found in the H group that practice hawker hunting type, especially due to the conformation of the compound eyes: i.e., contiguous compound eyes (little contiguous to broadly contiguous) that marks this group and this character.

Extended data fig. 6 illustrates an analysis of log shape ratio, a method to remove the size effect from a morphometric data set[27]. This allows to highlight several groupings (more or less by organs: eyes, head, thorax, legs) and oppositions of variables, such as the inverse relationship between the length and the thickness of the protibia. The size effect is always present and discriminates all fossil species including [Meg]. The shape of the compound eyes is less discriminating in this analysis.

This first preliminary study will make it possible to set up morpho-functional comparative methods including fossils and extant lineages by increasing the sampling, in the extant one and for the fossils that have their head preserved. A more complete analysis is in progress in this direction and will detail this new comparative approach, including phylogenetic considerations.

The selected morphometric characters are relevant to discrimine taxa and morpho-functional features in our dataset, including fossils and in particular *M. gracilipes*. This unique specimen is critically important for deducing the paleobiology of this lineage. The size variable played probably a functional role in the life traits of these organisms (to determine their ecological niche). It may be relevant to take their sizes into account in comparative morpho-functional approaches as demonstrated herein.

References

1. Nel, A., Fleck, G., Garrouste, R. Gand, G., Lapeyrie, J., Bybee, S.M. & Prokop, J. Revision of Permo-Carboniferous griffenflies (Insecta: Odonatoptera: Meganisoptera) based upon new species and redescription of selected poorly known taxa from Eurasia. *Palaeontographica* (A) **289**, 89–121 (2009).
2. Bechly, G. Morphologische Untersuchungen am Flügelgeäder der rezenten Libellen und deren Stammgruppenvertreter (Insecta; Pterygota; Odonata), unter besonderer

Berücksichtigung der Phylogenetischen Systematik und des Grundplanes der Odonata. *Petalura Special Volume* **2**, 1–402 (1996).

3. Meunier, F. Nouvelles recherches sur les insectes du terrain houiller de Commentry, Allier. *Ann. Paléont.* **4**, 125–152 (1909).
4. Handlirsch, A. Revision der paläozoischen Insekten. *Denkschr. Kaiserl. Akad. Wiss., Math.-Naturwiss. Kl.Wien* **96**, 511–592 (1919).
5. Carpentier, F. & Lejeune-Carpentier, M. Conformation de l'abdomen d'un insecte Protodonate du Stéphanien de Commentry. *Ann. Soc. Géol. Belgique* **72**, 317–326 (1949).
6. Carpentier, F. & Lejeune-Carpentier, M. Observations sur la morphologie des Meganeuridae (Insecta, Protodonata) du Stéphanien de Commentry. *Compt.-Rend. du 13ème Congr. Internat. Zool. Paris* **13**, 553–554 (1949).
7. Carpentier, F. & Lejeune-Carpentier, M. Structure du thorax des Meganeuridae (Protodonata). *Trans. 9th Internat. Congr. Entomol.* **1**, 161–164 (1952).
8. Carpentier, F. Sur une figure récente de *Meganeurula gracilipes* Handl. (Protodonate du Houiller). *Bull. Ann. Soc. Entomol. Belgique* **89**, 183-184 (1953).
9. Carpenter, F. M. Studies on Carboniferous insects from Commentry, France. Part 1. Introduction and families Protagriidae, Meganeuridae, and Campylopteridae. *Geol. Soc. Am. Bull.* **54**, 527–554 (1943).
10. Blanke, A., Schmitz, H., Patera, A. et al. Form-function relationships in dragonfly mandibles under an evolutionary perspective. *J. R. Soc. Interface* **14** (128) (20161038), 1–11 (2017).
11. Zessin, W. Zwei neue Insektenreste (Megasecoptera, Odonatoptera) aus dem Westfalium D (Oberkarbon) des Piesberges bei Osnabruck, Deutschland. *Virgo, Mitteilungsblatt des Entomologischen Vereins Mecklenburg* **9**, 37–45 (2006).

12. Zessin, W. Einige Aspekte zur Biologie paläozoischer Libellen (Odonatoptera). *Entomol. Generalis* **31**, 261–278 (2008).

13. Bechly, G., Brauckmann, C., Zessin, W. & Gröning, E. New results concerning the morphology of the most ancient dragonflies (Insecta: Odonatoptera) from the Namurian of Hagen-Vorhalle (Germany). *Z. Zool. Syst. Evolutionsforsch.* **39**, 209–226 (2001).

14. Brauckmann, C. & Zessin, W. Neue Meganeuridae aus dem Namurium von Hagen-Vorhalle (BDR) und die Phylogenie des Meganisoptera. *Deut. Entomol.e Z.* (N.F.) **36**, 177-215 (1989).

15. Brauckmann, C. Arachniden und Insekten aus dem Namurium von Hagen-Vorhalle (Ober-Karbon, West-Deutschland). *Veröffentlichungen aus dem Fuhlrott-Mus. Wuppertal* **1**, 1-275 (1991).

16. Hakim, Z.M. Comparative anatomy of the head capsules of adult Odonata. *Ann. Entomol. Soc. Am.* **57**, 267–278 (1964).

17. Fleck, G. La phylogénie des Odonata Anisoptera. Analyse de nouveaux caractères des structures céphaliques (Insecta). Diplôme d’Etude Approfondie, Systématique Animale et Végétale, Laboratoire d’Entomologie, Muséum National d’Histoire Naturelle, Paris, 1–31 (1996).

18. Petrulevičius, J. F. & Gutiérrez, P. R. New basal Odonatoptera (Insecta) from the lower Carboniferous (Serpukhovian) of Argentina. *Arquivos Entomolóxicos* **16**, 341-358 (2016).

19. Nel, A., Bechly, G., Prokop, J., Béthoux, O. & Fleck, G. Systematics and evolution of Paleozoic and Mesozoic damselfly-like Odonatoptera of the ‘Protozygopteran’ grade. *J. Paleont.* **86**, 81–104 (2012).

20. Jolliffe, I. T. Principal Component Analysis. Springer Series in Statistics (2002).

21. Brauckmann, C., Koch, L. & Kemper, M. Spinnentiere (Arachnida) und Insekten aus den Vorhalle-Schichten (Namurium B; Ober-Karbon) von Hagen-Vorhalle (West-Deutschland). *Geol. Paläont. Westf., Westf. Mus. Naturk*. **3**, 1–132 (1985).
22. Fleck, G. & Nel, A. The first isophlebioid dragonfly (Odonata: Isophlebioptera: Campterophlebiidae) from the Mesozoic of China. *Palaeontology* **45**, 1123–1136 (2002).
23. Mitteroecker, P. & Huttegger, S. M. The concept of morphospaces in evolutionary and developmental biology: mathematics and metaphors. *Biol. Theory* **4**, 54–67 (2009).
24. Chartier, M. et al. The floral morphospace—a modern comparative approach to study angiosperm evolution. *New Phytol*. **204**, 841–853 (2014).
25. Chessel, D. et al. The ADE4 package-I- One-table methods. R News **4**, 5–10 (2004).
26. Dray, S. & Dufour, A. B. The ADE4 package: implementing the duality diagram for ecologists. *J. Stat. Softw*. **22**, 1–20 (2007).
27. Darroch, J. N. & Mosimann, J. E. Canonical and principal components of shape. *Biometrika* **72**, 241–252 (1985).
28. Klingenberg, C. P. Size, shape, and form: concepts of allometry in geometric morphometrics. *Rev. Gen. Evol*. **226**, 113–137 (2016).
29. Oksanen J. Multivariate analysis of ecological communities in R: Vegan package (https://cran.r-project.org/web/packages/vegan/) (2015).
30. Wootton, R. J. The functional morphology of the wings in Odonata. *Advanced in Odonatol*. **5**, 153–169 (1991).
31. Johansson, F., Söderquist, M., Bokma, F. Insect wing shape evolution: independent effects of migratory and mate guarding flight on dragonfly wings. *Biological Journal of the Linnean Society* **97**, 362–372 (2009).

32. Bomphrey, R.J., Nakata, T., Henningsson, P., Lin, H.-T. Flight of the dragonflies and damselflies. *Phil. Trans. R. Soc.* (B) **371**, 20150389. (2016).

33. Garrouste, R. et al. Insect mimicry of plants dates back to the Permian. *Nature Communications* **7** (13735), 1–6 (2016).

**Extended Data Figure 1 | *Meganeurula selysii* (Brongniart, 1893), holotype MNHN R52939, head and prothorax**. ventral view showing the false mandibles carved in matrix (photograph Gaelle Doitteau, e-recolnat Project, MNHN). Scale bar, 10 mm.

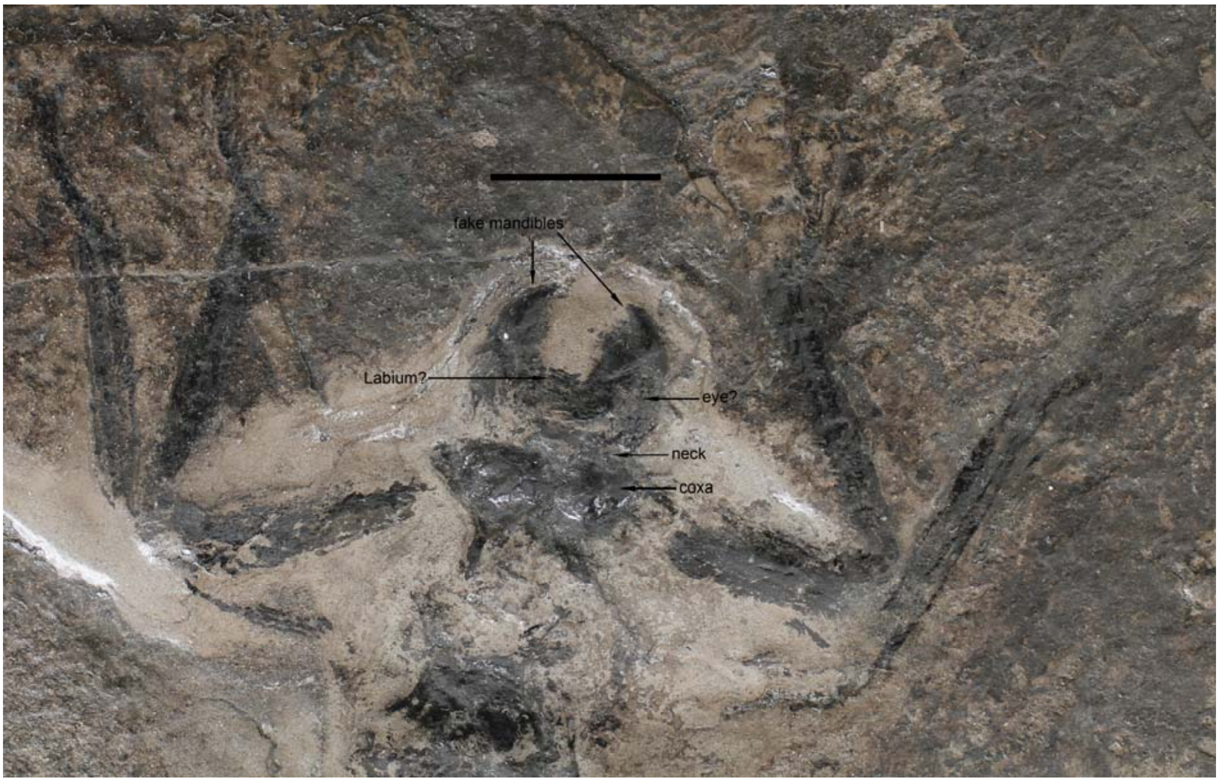


**Extended Data Figure 2 | Photographs of heads of extant hawker dragonflies. a**, *Aeshna isoceles* (Müller, 1767) (Aeshnidae, Var, France); **b**, *Micrathyria* sp. (Libellulidae, French Guyana); **c-d**, *Gynacantha klagesi* Williamson, 1923 (Aeshnidae, same male specimen, French Guiana); **e**, *Onychogomphus* sp. (Gomphidae, Var, France); **f**, *Mecistogaster amalia* (Burmeister, 1839) (Pseudostigmatidae, Argentina) (MNHN collection). **a-e** Epiproctophora, **f** Zygoptera. **e-f** perchers, others are hawkers.

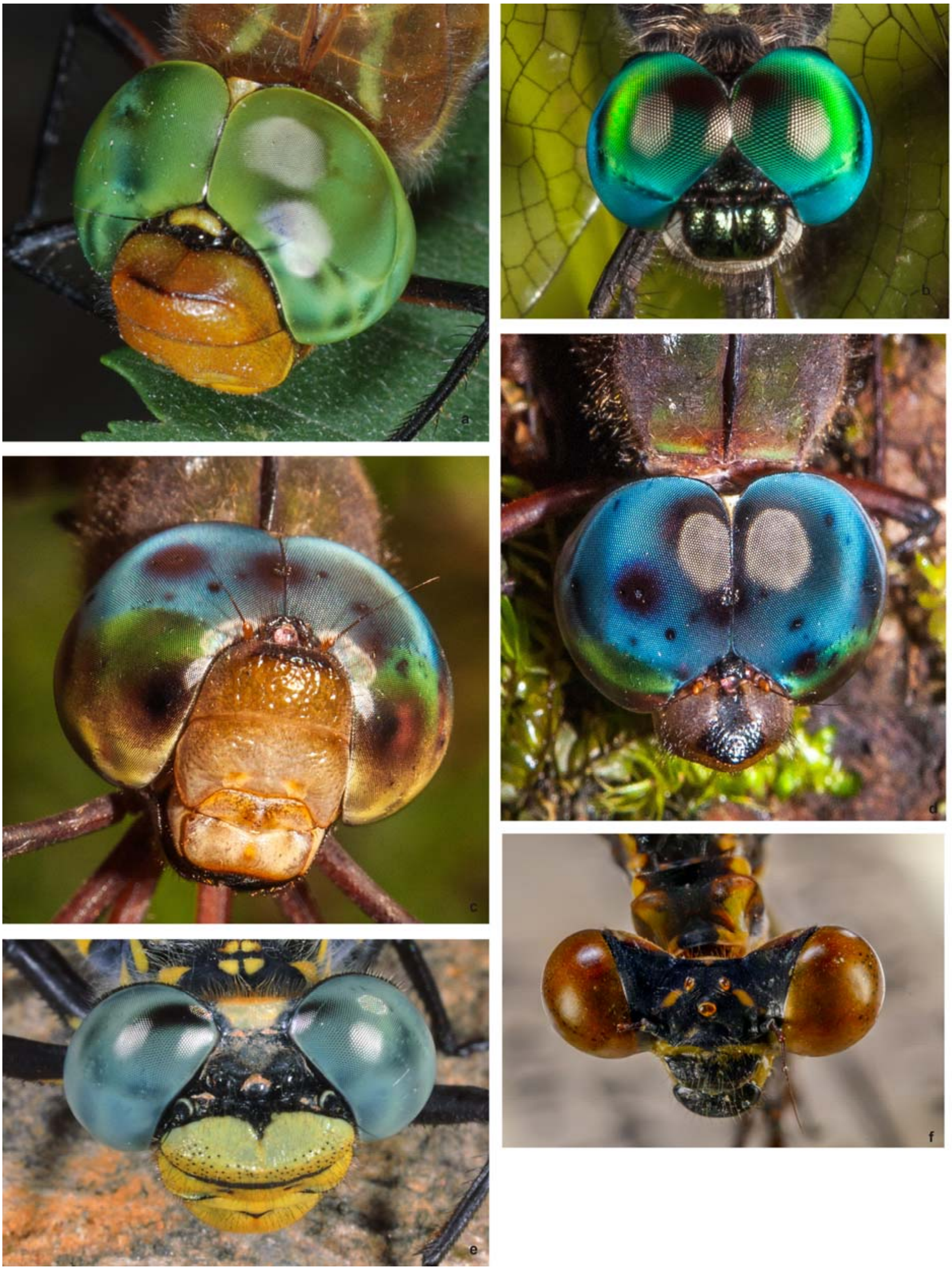
a
b
c
d
e
f

**Extended Data Figure 3 | Diagram for captures of morphometric measurements.**

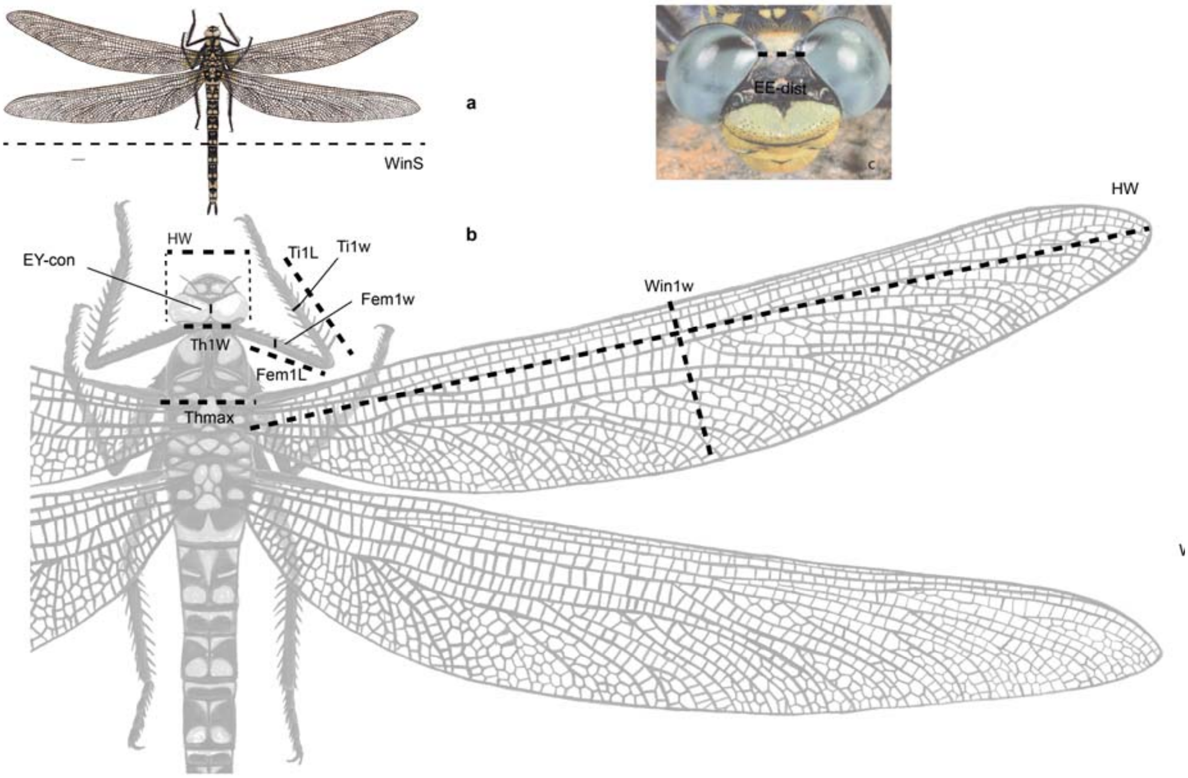

**Extended Data Figure 4 | PCA 1: multivariable analysis on unprocessed raw data**. 12 morphological variables, 23 taxa; **a**, projection on axes 1-2 taxon plate, grouped by families; **b**, circle of correlations; **c**, graph of the eigenvalues (inertia of the axes); **d**, projection on axis 2-3 of the taxa grouped by type of hunting behavior; P: percher, H: hawker. Name of variables and taxa in text. Fossils taxa with frame. Reconstruction of *Meganeurites gracilipes*.

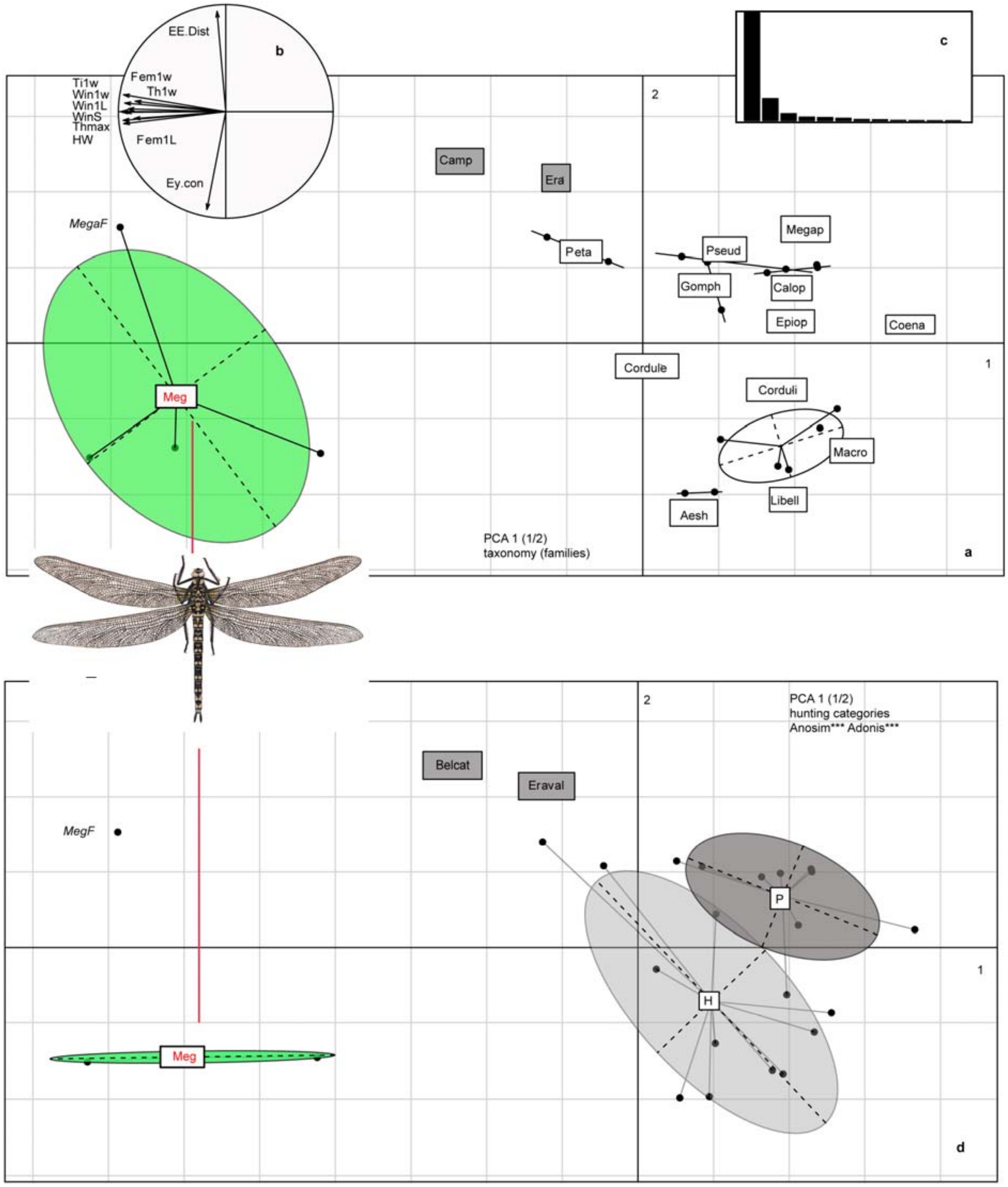

**Extended Data Figure 5 | PCA 1: multivariable analysis on unprocessed raw data**. 12 morphological variables, 23 taxa; **a**, projection on axes 2-3; **b**, correlations circle (variables); **c**, graph of the eigenvalues (inertia of the axes). Name of variables and taxa in the text. Fossils taxa with frame. Reconstruction of *Meganeurites gracilipes*.

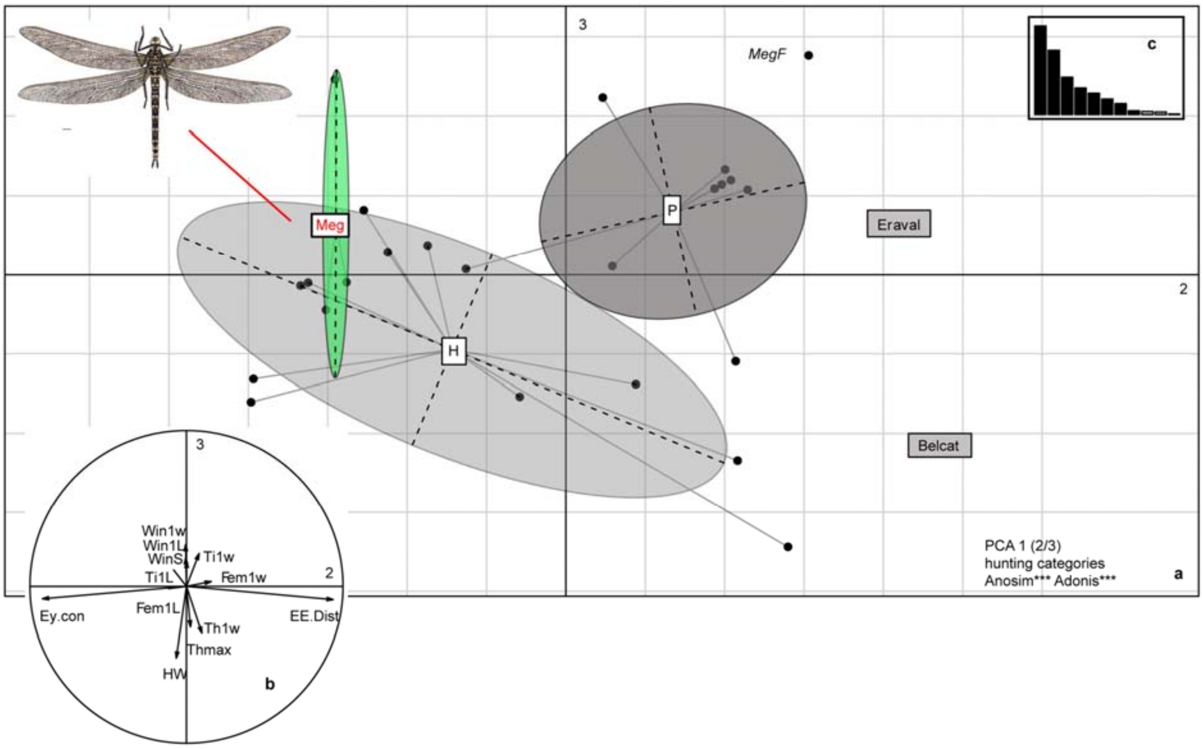

**Extended Data Figure 6 | PCA 2: multivariable analysis on log shape ratio**. 12 morphological variables, 26 taxa; **a**, projection on axes 1-2; **b**, circle of correlations circle (variables); **c**, graph of the eigenvalues (inertia of the axes). Name of variables and taxa in the text. Fossils taxa with frame. Reconstruction of *Meganeurites gracilipes*.

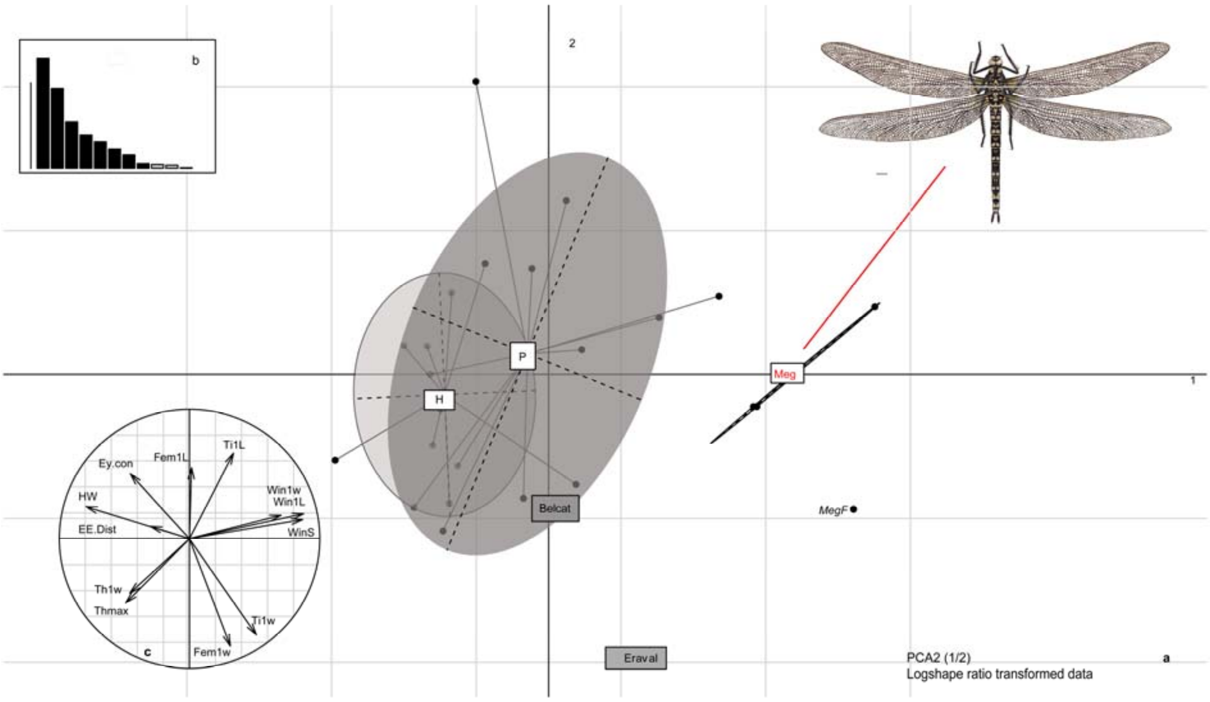

Supplement: Supplementary file 1 — Supplementary Material [file 41598_2018_30629_MOESM1_ESM.pdf]
